# Daily Performance of Adolescents with Executive Function Deficits: An Empirical Study Using a Complex-Cooking Task

**DOI:** 10.1155/2020/3051809

**Published:** 2020-02-06

**Authors:** Yael Fogel, Sara Rosenblum, Renana Hirsh, Mathilde Chevignard, Naomi Josman

**Affiliations:** ^1^The Laboratory of Complex Human Activity and Participation (CHAP), Department of Occupational Therapy, Faculty of Social Welfare and Health Sciences, University of Haifa, Mount Carmel 3498838, Israel; ^2^Rehabilitation Department for Children and Adolescents with Acquired Neurological Injury, And Outreach Team for Children and Adolescents with Acquired Brain Injury, Saint-Maurice Hospitals, 14 rue du Val d'Osne, 94410 Saint-Maurice, France; ^3^Sorbonne Université, Laboratoire d'Imagerie Biomédicale, LIB, 75006 Paris, France

## Abstract

**Purpose:**

To characterize and analyze the performance of adolescents with executive function deficits through the Children's Cooking Task (CCT) as a performance-based complex ecological assessment.

**Methods:**

Participants were 41 adolescents (aged 10–14 years) with normal intellectual function and executive function deficit profiles based on the Behavior Rating Inventory of Executive Function (BRIEF) parent reports and self-reports (BRIEF-SR) and the WebNeuro and 40 controls with typical development matched by age and gender. Participants in both groups performed the CCT, an ecological standardized complex task.

**Results:**

Significant group differences were found for all CCT outcome measures (total number of errors, task duration, and all qualitative rating variables). Significant correlations were found among children with executive function deficit profiles between the CCT performance duration and total number of errors and the BRIEF-SR subscale score. Two separate discriminant function analyses described primarily by the CCT correctly classified the study groups.

**Conclusion:**

The poor performance of adolescents with executive function deficit profiles exhibited through the standardized complex task, as well as the relationships with their executive functions, supplies better insight about their daily confrontations. Identifying how they perform may lead to development of focused interventions to improve these adolescents' daily performance, participation, and wellbeing.

## 1. Introduction

Executive functions (EF) are control functions people use to implement or execute a task in order to face new situations. These multidimensional cognitive constructs are necessary for goal-directed and problem-solving behavior in all aspects of life, whether academic, vocational, or social [1]. According to theoretical models, EF are essential in the cognitive development of children and adolescents and are involved in learning processes [2, 3] and everyday occupations [4]. The many EF definitions vary from one researcher to another; however, most researchers agree that the foundational components of EF are inhibition, working memory, and shifting or cognitive flexibility [5, 6].

The EF components integrate to allow people to perform complex, novel, and dynamic occupations successfully [7]. When EF develop normally, behavior is adapted according to the changing environments. However, when EF deficiencies are present, significant delay may be observed in subsequent cognitive, social, and academic development [8]. The developmental transitions from childhood to adolescence and then to adulthood involve greater use of EF [9]. Based on extensive recent research about EF and their impact on learning, daily functioning, and behavior, EF may be most relevant for understanding the reason for deficits or success in activity performance and participation abilities among children and adolescents with neurodevelopmental disorders [10].

A profile of EF deficits (EFD) characterizes a heterogeneous group of children and adolescents who struggle in their daily functioning, including in the school environment, with or without diagnoses of neurodevelopmental disorders such as attention deficit hyperactive disorder [11], developmental coordination disorder (DCD) [12], or specific learning disabilities [13].

Because EF are important for coping with and managing daily life challenges, it is important, in addition to the standard neuropsychological testing, to examine them within the context of challenging activities and everyday life contexts (i.e., ecological assessment) to obtain a complete picture of a person's functioning [14]. Barkley [15] described *ecological validity* as the extent to which performance evaluated in a laboratory represents the actual behaviors of interest as they occur in natural settings. Ecological validity in relation to neuropsychological assessment has two requirements: first, the demands of the test and the testing conditions resemble demands in the child's everyday world, or *verisimilitude*; second, performance of the test predicts some aspects of the child's functioning on a day-to-day basis, or *veridicality* [4, 16].

Two main types of ecological assessment of EF have been developed and studied [17]: (1) rating scales/questionnaires evaluating EF in common daily situations (at home or in school), such as the Behavior Rating Inventory of Executive Functions (BRIEF) [18–20] or the Dysexecutive Questionnaire for Children [21] and (2) performance-based assessments. Functional assessments may include (a) functional >“paper and pencil” tasks simulating situations close to daily life tasks but performed in an office, such as the Behavioral Assessment of Dysexecutive Syndrome for Children (BADS-C) [21] or (b) real-life situation tasks with observation of actual performance in a natural environment, such as the Do-Eat [22, 23], the Weekly Calendar Planning Assessment [24], or the Children's Cooking Task (CCT) [25].

Chevignard et al. [25] developed the CCT to provide an ecologically valid assessment in a real-life, open-ended setting. The assessment, performed and rated by occupational therapists in clinical practice, proved feasible in detecting and characterizing (even mild) EFD in children with traumatic brain injury [26] and DCD [27]. The unique error analysis used in the CCT was initially developed using Lezak et al.'s [28] cognitive model, in which an execution error may reflect dysfunction in volition, planning, goal direction, or task monitoring. Additionally, Schwartz et al.'s model of action disorganization syndrome was used to precisely describe errors. This allowed developing a two-stage error rating. In the first step, purely descriptive errors (e.g., omissions or additions) are rated without reference to how or why they occurred. In the second step, each error is reconsidered following a “neuropsychological” error rating, allowing description of the reasons each error occurred (e.g., control or context neglect errors).

The initial Adult Cooking Task [29, 30] and the subsequently developed and adapted children's version, the CCT [25, 26], were found to be highly sensitive to executive dysfunction. There is a great need to take these performance-based assessments a step forward to provide more accurate and sensitive assessment of everyday functional activities that consider the person's environment [31]. Performance-based assessments such as the CCT have the potential to detect EF difficulties because its tasks capture the complexity of real-life performance [32–34]. These kinds of assessments allow clinicians to not only evaluate the testing outcome, but also analyze the process, including the client's general approach, strategy use, online control, and observable problem-solving behavior [14].

The main aim of the present study was to characterize and analyze the performance of adolescents with EFD through the CCT as a performance-based complex ecological assessment. The results can enhance the utility of this novel ecological performance-based measure for use with adolescents with and without EFD profiles. As such, we proposed the following hypotheses:

- Significant differences will be found between adolescents with EFD and those with typical development (TD) in performance of a complex task, as evaluated by the CCT (i.e., in the total number and types of errors, task duration, and qualitative *rating* variables)
- Significant correlations will be found between the total number of errors and task duration in the CCT and EF measures (BRIEF-SR and WebNeuro) in each group separately
- The number of errors on the CCT will predict group membership (EFD or TD)

## 2. Method

### 2.1. Instruments to Examine Inclusion Criteria

#### 2.1.1. Demographic Questionnaire

The demographic questionnaire includes information about age, gender, mothers' and father's education, socioeconomic status, and diagnosis.

#### 2.1.2. BRIEF Parent Version [18]

The BRIEF parent version was used to measure EF performance in everyday life and to determine group membership. This version includes 86 questions in five cognitive subscales (initiation, working memory, plan-organize, organization of materials, and monitoring) and three behavioral subscales (inhibit, shift, and emotional control). These eight subscales form two composite indices (MI and BRI) and one global executive composite (GEC) score, with a mean of 50 and a standard deviation of 10. The results are presented in *t*-scores (with higher scores indicating worse performance). A *t*-score of 65 or above is considered in the clinical range. Mean internal consistency ranges from .82 to .98, and test-retest reliability ranges from .72 to .84.

#### 2.1.3. WISC-R [35], Hebrew Version

The WISC-R assesses verbal and nonverbal capacities to evaluate intellectual functioning. We utilized its vocabulary and block design subtests to assess these capabilities, define participants' profiles (presenting with at least average cognitive abilities), and rule out participants with possible intellectual disabilities. The mean score in each subtest is 10. Sattler reported that the two-subtest combination of vocabulary and block design has a strong correlation with the WISC-IV full-scale IQ (*r* = .92) and adequate test-retest reliability (*r* = .87) [36]. We chose these tests also because they are commonly used to assess intellectual function in evaluation studies in a population of children aged 10 years or older with complex neurodevelopmental disabilities [37, 38]. An occupational therapist performed the assessment, and a psychologist coded and interpreted them.

### 2.2. Measures to Characterize EFD Profile

#### 2.2.1. BRIEF-SR [20]

The BRIEF-SR is a self-report consisting of a reliable and valid questionnaire used to assess EF in 11- to 18-year-olds with a reading level of at least fifth grade. The current study included participants from age 10, in fourth grade with an appropriate reading level and no difficulties completing the questionnaire. Notably, there is more evidence for using the BRIEF-SR beyond the age range [39, 40].

The BRIEF-SR includes 80 statements in four subdomains (inhibition, shifting, emotional control, and monitoring) of the BRI and the four subdomains of the MI (working memory, planning/organization, organization of materials, and task completion). The BRI and MI scores are combined to constitute the overall GEC score. A GEC *t*-score of 50 represents the average standard score. A standard *t*-score of 65 or greater is considered in the clinical range. Test-retest reliability was found to be .84 for the BRI and .87 for the MI.

Internal consistency for the GEC, BRI, and MI in the current study was *α* = 0.95. It seemed that the questionnaire's reliability was not affected by participants at ages 10 to 11 years. Thus, the score was calculated for them as children at age 11 years.

#### 2.2.2. WebNeuro [11]

WebNeuro is a computerized neuropsychological screening tool designed to emphasize cognitive function and its effects in different populations. Its several versions allow repeated measurement at points throughout the intervention, building a person's profile over time. WebNeuro taps the sensorimotor, memory, executive, attention, and emotion perception (social cognition) domains of cognitive function. The WebNeuro was used in this study to characterize the participant EFD profiles as an objective assessment alongside self-reported or observation-type evaluations. As such, it strengthened the population's characterization. For most tests, WebNeuro's automated software scores the responses. Each study participant's performance on each aspect of each test (reaction time, accuracy, etc.) is expressed as a *z*-score relative to the normative mean of 0, with positive *z*-scores reflecting better than average performance, and negative values reflecting poorer than average performance. The validity coefficient between IntegNeuro (a non-Internet-based computerized cognitive assessment battery) and WebNeuro factors and overall performance scores has been found to be between .56 and .86, and the validity coefficient between critical scores for each test range from .45 to .87. All study participants completed the memory, attention, and executive domains of the WebNeuro.

### 2.3. Primary Outcome Measure

#### 2.3.1. CCT

The CCT is a performance-based evaluation developed by Chevignard et al. [25] to assess EF and multitasking abilities. The CCT is designed for children and adolescents aged 8 to 18 years. However, depending on their autonomy and independence, teenagers aged 16 to 18 years can also be assessed using the adult version of the test. Participants are asked to prepare two simple recipes (a chocolate cake and a fruit cocktail).

The examiner reads written instructions to the child and checks that they are fully understood. The instructions are available to the child throughout the task. Participants are informed that they must pretend to be completely alone to prepare a surprise for their family. An examiner only intervenes in case of “fatal” mistakes or dead ends to avoid complete failure or to put the child back on track to finish the task. All participant actions and comments are recorded and analyzed later. The assessment for our study took place in a therapeutic kitchen within the occupational therapy center, and the same setting was used for all participants. Ingredients and utensils laid out on the table included semantic and morphological distractors. Given a folder with six recipes presented in the same format (i.e., title, illustrated list of ingredients, and numbered illustrated preparation steps), each participant was expected to find the two correct recipes (chocolate cake and fruit cocktail).

The CCT is categorized as a “real-life” situation and performance-based assessment because it is not performed in a neuropsychology office or an occupational therapy room, but in a real kitchen (the child's actual kitchen or one the child does not know) using real materials and ingredients. It entails the child manipulating real-life instruments, ingredients, and so forth and performing an activity that is or could be part of everyday life. In many instances, measuring how much the children questioned or asked for help assisted in realizing how impaired they were in performing an (everyday life) activity on their own (without help) and how much they had to rely on adult guidance to finish the task.

The CCT analysis consists of classifying and quantifying the errors observed. They are classified at two levels (the appendix) [25]: a *descriptive* level and a *neuropsychological* level [41]. The descriptive level includes errors of omission, addition, commentary-question, substitution-sequence, and estimation. In this study, the examiner counted the number of each type of error and summarized the total. Then, to interpret the cause of the mistakes, the errors were classified according to neuropsychological levels. There are six error types for this level: control, context neglect, environmental adherence, purposeless action and displacement, dependency, and inappropriate behavior (defined and described in the appendix).

In this study, a qualitative rating of the task was conducted, taking into account the time required to complete the task (in minutes), as well as three criteria (rated yes or no): ability to reach the task goal (for the subject to prepare both an edible cake and a fruit cocktail), onset of dangerous behavior (e.g., the child wanted to use the oven unsupervised or used a knife found in the kitchen), and required adult intervention to prevent complete task failure (e.g., the child forgot to perform part of the instructions, did not monitor cooking time, or asked for help). A clinical cut-off score was set for each error type and for task duration using the mean number of errors and time in the control group plus two standard deviations. Participants performing beyond this cut-off were considered in the clinical range.

The CCT manual, which was developed in French and translated into English [41] (including psychometric properties), was published in two articles [25, 26]. Internal consistency Cronbach's alpha = .86 (*n* = 25 traumatic brain injury) [26]; interrater reliability for the total number of errors was .96 and for other error types ranged from .70 to .99 [25]. Test-retest reliability was .89 for the total number of errors and from .46 to .90 for other types of errors, .94 for task duration, .63 for goal achievement, and .53 for need for adult intervention [17].

The authors provided permission to translate the CCT into Hebrew for use in this study, following a forward-and-backward translation. Content validity for using the assessment in Israel was based on a pilot study with five children and a focus group of seven expert occupational therapists from the University of Haifa's Department of Occupational Therapy. Additionally, two occupational therapists trained in the assessments scored five children who performed the assessment. The results showed high-level reliability. Internal consistency of the Hebrew CCT is high (*α* = .81) and test-retest reliability is moderate for the total number of errors (intraclass correlation coefficient = .65) [42]. Moderate concurrent validity of the CCT assessment was found with the parent-rated BRIEF and WebNeuro [11, 42].

### 2.4. Participants

Study participants were recruited through published community advertisements aimed towards young adolescents (aged 10–14 years) with and without daily functioning difficulties. We determined sample size using G-Power software, considering the medium effect size of 0.25, power = 90, and *α* = 0.05 [43, 44].

Respondents to the study advertisements who had known psychiatric or emotional disorders, autistic spectrum disorders, physical disabilities, or neurological diseases were excluded from the study. The remaining were invited for examination for the inclusion criteria. Specifically, parents completed the BRIEF [18] and a demographic questionnaire, and adolescents performed two subtests from the Wechsler Intelligence Scale for Children (WISC-R) [35], with a score higher than 5 required for inclusion. All 81 respondents met the criteria, and they and their parents were invited to participate in the study.

The participants were divided into two groups—one, 41 adolescents with EFD, the other, 40 adolescents with TD—according to the BRIEF scores. Specifically, inclusion in the research (EFD) group required the adolescents to score a minimum score of 65 (outside normal range) on the metacognition (MI) or behavioral regulation (BRI) composite indices of the parents' BRIEF reports. Inclusion in the control (TD) group, matched to the group with EFD by the adolescents' age and gender, required index scores in the normal range (<65) according to the parents' BRIEF reports in the BRI or the MI.

Then, the participants who were found appropriate completed the BRIEF-SR questionnaire [20], the WebNeuro computerized assessment [34] for characterization as participants with or without EFD profiles, and the CCT assessment for the outcome measure.

The rationale for choosing these measures was based on the literature recommendations to use rating measures and performance-based measures because they assess different aspects of executive function and together provide an indication of how well or poorly an individual responds to adverse conditions [45].

### 2.5. Procedure

The University of Haifa Ethics Committee approved this study. The participants were recruited through published community advertisements aimed towards adolescents aged 10 to 14 years with and without everyday functioning difficulties and with or without formal diagnoses. The adolescents completed two subtests from the WISC-R for inclusion criteria, and their parents completed demographic and BRIEF questionnaires to determine inclusion in the study. Participants who met the inclusion criteria were invited for a one-time meeting. After all participants and their parents signed informed consent forms, each participant then completed the BRIEF-SR questionnaire and the WebNeuro computerized assessment for characterization as participants with or without EFD profiles and the CCT assessment as the outcome measure. An expert occupational therapist who was blinded to the participant's group status (EFD or TD) administrated and, except for the WISC-R, analyzed all assessments.

### 2.6. Data Analysis

Data processing with SPSS-21 provided descriptive statistics (mean, standard deviation, and percentages) to describe the study participants' main variables. Those that referenced the CCT are combined results for the two recipes (making the cake and the fruit cocktail). Because the CCT errors were not normally distributed, a Mann-Whitney test was conducted to examine differences between groups in the CCT assessment. Spearman correlations were used to examine the correlation analyses between the CCT and the BRIEF-SR questionnaire and the WebNeuro. The effect sizes are represented by partial eta squared, with *η*_*p*_^2^ < .04 considered a minimum effect size representing a clinically significant effect, .04 < *η*_*p*_^2^ < .25 considered a moderate effect, and *η*_*p*_^2^ > .25 considered a strong effect size [46]. Fisher's linear discriminant function analyses were conducted to determine which CCT variables best predicted group membership.

## 3. Results

Overall, 41 participants met the inclusion criteria for the EFD group, and 40 matched TD adolescents constituted the control group. Demographic and functional characteristics of both groups are presented in Table 1.

Both groups (EFD and TD) were within the average range (10) for the two WISC-R subtests. However, significant differences were found between the two groups, with a lower mean in the EFD study group ([*F*(2, 78) = 11.30, *p* < .001, *η*_*p*_^2^ = .23]) in the parent-report BRIEF indices: BRI (*t*_(79)_ = 11.73, *p* < .001), MI (*t*_(79)_ = 17.1, *p* < .001), and GEC (*t*_(79)_ = 19.44, *p* < .001); the self-report BRIEF indices: BRI (*t*_(79)_ = 5.27, *p* < .001), MI (*t*_(79)_ = 7.08, *p* < .001), and overall GEC score (*t*_(79)_ = 6.14, *p* < .001); and the WebNeuro [*F*(6, 74) = 11.84, *p* < .001, *η*_*p*_^2^ = .49]. The participant inclusion criteria and the additional measure to characterize EFD profiles are presented in Table 2.

**Hypothesis 1 A (differences between groups (CCT)).**

Significant differences were found between the groups in the CCT assessment scores.  Table 3 shows that the adolescents with TD performed significantly better than did the adolescents with EFD in total number of errors, all error types, task duration, and qualitative rating (i.e., goal achievement, dangerous behavior, and required adult intervention). The table also shows that, except for *omission*, all descriptive- and neuropsychological-level errors among the group with EFD were more than two standard deviations above the group with TD, based on the CCT clinical cut-off score.

**Hypothesis 2 B (correlations between the CCT total number of errors and task duration and the EF profile measures (BRIEF-SR subscales and WebNeuro)).**

Spearman correlations between the total number of errors and task duration (CCT) and the EF measures (BRIEF-SR and WebNeuro) were established in each group separately. A medium positive correlation was found between the BRIEF-SR subscales plan/organization (*r* = .31, *p* < .05) and task duration. No other significant correlations were found in the EDF and TD groups.

**Hypothesis 3 C (predictive validity of error types).**

Discriminate function was found for group classification of participants in the descriptive (*Λ* = .32, *p* < .001) and neuropsychological (*Λ* = .42, *p* < .001) analyses of the CCT. As presented in Table 3, all error types contributed considerably to group classifications. The variables providing the greatest contribution to group membership were *estimation error* from the descriptive analysis (loading = .81) and *control error* from the neuropsychological analyses (loading = .77). Values for all error types are presented in Table 4. Based on these functions for the descriptive analysis of the CCT, 91.4% of the study participants were correctly classified into their respective groups (specifically, 88.0% of participants in the group with EFD and 95.0% in the TD group). A kappa value of .83 (*p* < .001) was calculated, demonstrating that group classification did not occur by chance. For the neuropsychological analyses, 88.9% of study participants were correctly classified into their respective groups (i.e., 82.9% of participants in the group with EFD and 95.0% in the TD group). A kappa value of .77 (*p* < .001) was calculated, demonstrating that group classification did not occur by chance.

## 4. Discussion

The main aim of this study was to characterize and analyze performance of adolescents with EFD through a performance-based complex ecological assessment—the CCT assessment—and to explore the relationships between the CCT and EF measures. The results enhance the utility of this novel ecological EF measure for use with adolescents with EFD.

Results show that participants with EFD made significantly more errors than those with TD (Hypothesis A). The types of errors and the qualitative rating criteria distinguished adolescents with EFD from those with TD. Our results are similar to findings from previous studies indicating that adolescents with executive dysfunctions can be impaired when performing complex activities of daily living [10, 25, 26, 41]. For example, the adolescents with EFD in this study did not reach the assessment goal as efficiently as did their peers with TD. The group with EFD required significantly more adult assistance, exhibited significantly more dangerous behaviors, and needed significantly more time to complete the task. These results also align with previous CCT assessment findings among children with developmental dyspraxia [27] or traumatic brain injury [25, 26] and strengthen the CCT's ability to discriminate between different health condition groups compared to typical development groups, as reported in those three studies.

That the highest average errors were of the *addition* and *commentary-question* types may suggest that the adolescents' behavior during the task reflected their daily conduct in complex tasks. Most adolescents in the group with EFD performed unnecessary moves and steps (*addition*), as well as asked questions and commented (*commentary-question*), although they had been explicitly told to act as though they were alone. The neuropsychological-level analysis shows a considerable number of errors in participants' *context neglect*, *control*, *environmental adherence*, *purposeless action*, and *dependency*. Almost half of the participants in the group with EFD required adult intervention to prevent task failure (vs. 7% of the group with TD). These results indicate important monitoring difficulties during a complex task, leading to numerous unnecessary or unrelated actions and increased need for external help and cues, thus increasing the time needed to complete the task [26]. The literature described children and adolescents with EFD profiles as disorganized, lacking initiative, and forgetful. They require frequent encouragement and cues when expected to do more than one thing at a time. Because they have difficulty starting assignments, they may be described as lazy or procrastinating. They struggle to shift between activities, may persevere on one task until completed, and have difficulty prioritizing important tasks, managing time, and meeting deadlines. Planning challenges them because they tend to focus only on the present [1]. These characteristics are reflected in their EFD profile (as parents reported through the BRIEF), and the children's performance (number of errors) on the CCT observed in the current study strengthens that profile. Such difficulties in daily performance are a main reason for referral to therapy services such as occupational therapy.

The correlations found between the EF measures and total number of errors and task duration (Hypothesis B) are few and medium (one correlation), similar to the literature, which documented that performance-based and questionnaire-based EF measures assess different underlying mental constructs [45, 47]. Performance-based measures of executive function occur under maximal or optimal performance situations and assess the processing efficiency of cognitive abilities under very structured conditions. Rating measures of executive function occur under typical performance situations and assess the extent to which individuals accomplish goal pursuits under unstructured conditions. The former are “supervisory,” and the latter involve “executive” control [47].

Although only one correlation was found between the CCT and the EF measures—specifically, a medium positive correlation between the BRIEF-SR subscales plan/organization and task duration—it may strengthen the evidence for presence of EF in everyday tasks and their possible implications on performance and participation. We measured the time needed to complete the task because organizing daily activities and tasks and their execution time are important keys to efficient time usage and daily expressions of how personal temporal abilities affect an individual's procedure over time. These abilities are required for independent work and long-term projects [13]. The gaps between individuals' abilities and environmental requirements will manifest in the individuals' manner of organizing activities in time and may influence their participation and success in life events [48]. Zentall et al. [49] linked the ability to plan and execute an activity within a limited time, place an object where it can be easily located, and plan how to execute the activity as a construct of time and space organization. *Addition errors*, one of the most common errors among adolescents with EF in our study, are defined as any action or sequence of actions unnecessary to task completion (e.g., adding to the instructions or utilizing distractors, opening and closing doors, and taking objects and returning them to their place without using them). These errors extend the task duration and, of course, do not help achieve the goal. They may also demonstrate organizational deficits in space that affect performance. Similar findings were found in published studies of adults with acquired brain injury [30] using the Cooking Task: the task duration did not correlate to processing speed, but mostly to the number of errors. Indeed, the more errors or additional actions participants made, the more corrective actions they needed to perform, and this slowed their overall task performance. Prior studies showed that functional difficulties among adolescents with EFD lead to more time needed to complete a task. Reasons include slow information processing and difficulty automating operations [50]. Clinicians might take into account this component and consider it in therapy sessions, for example, to create useful strategies to deal with challenging daily activities that involve plan/organization skills.

Discriminant function analysis revealed that the CCT classified a high percentage of the adolescents into their appropriate groups (Hypothesis C). However, almost all error types contributed to the assessment of function and thus should be considered during intervention. This confirms the high internal consistency found for the Children's [26] and the Adults' [41] Cooking Tasks.

Performance-based tests applied in a structured setting can provide valuable information about optimal performance and can therefore predict performance in similar settings, such as school tasks in the classroom. However, tests of this kind cannot accurately predict performance in goal-directed behavior in real-world settings, for instance, in complex social situations. Everyday settings are less structured; the level of demand varies and there is no instruction from the evaluator. Furthermore, performance is estimated by the self or by other informants, a circumstance that does not guarantee maximum accuracy [45]. Combining the use of performance-based tests and rating scales in the comprehensive assessment of executive functioning may be the best way to assess daily functions among young adolescents with EFD [47, 51–53].

The CCT is classified as a real-life situation task with observation of actual performance in a natural environment because it includes activities resembling everyday functions [54]. The task is easily adapted to children and adolescents, and the participants enjoy performing it. Moreover, open-ended tasks such as those in the CCT add meaningful information to the participants' everyday adaptive functioning. Observing the task performance allows the therapist to promote the self-awareness process by identifying execution errors to assist in developing self-monitoring skills and to create effective strategies and training for transferring and generalizing to additional activities and different environments [55].

Despite the effective information obtained from the CCT, this study had several limitations. Importantly, it was not possible to access the medical files and data (e.g., tests used, exact diagnosis retained, or comorbidities) of participants in the group with EFD to formally verify the diagnoses they reported. Because some participants had no formal diagnoses, the study results cannot be applied to the overall population with conditions associated with EFD. Additionally, despite the clear difference between groups in levels of errors, as suggested by the Mann-Whitney tests, we are aware of a discriminant function analysis to predict group affiliation in a multivariate model due to the normality assumption at the core of this type of analysis.

Another limitation was that the potential influence of the examiner's presence in the kitchen cannot be removed from the participant's use of independent self-regulatory behaviors; participants, therefore, were not observed in a fully natural environment [25].

Future studies should attempt to confirm this study's findings with larger representative samples and to examine the CCT as an outcome measure after treatment [56]. The studies could use recipes (of the same difficulty level) other than those used in the current study to maintain this outcome measure's novelty. Future studies should also examine the cognitive strategies participants use during the task to learn more about efficient and inefficient functioning for the therapy intervention.

In conclusion, this study's findings can help researchers and clinicians characterize and analyze the performance of adolescents with EFD. The results suggest evidence for the utility of the CCT as an assessment tool, examining everyday EF in both clinical and research settings. The findings allow clinicians to choose the CCT when it suits the child, in order to create an evaluation process that incorporates accurate assessment tools.

## Figures and Tables

**Table 1:** Participants' demographic characteristics.

	EFD group (*n* = 41)		TD group (*n* = 40)		*t*	*p*
	*M* (SD)	Range	*M* (SD)	Range		
Age (years)	11.88 (1.08)	10.08–14.05	12.19 (1.08)	10.00–14.75	-1.29	.2
	Frequency (%)		Frequency (%)		*χ* ^2^	*p*
Gender						
Boys	29 (70.7)		28 (70.0)		0.0	.94
Mothers' education						
Academic	34 (82.9)		37 (92.5)		1.71	.19
Fathers' education						
Academic	30 (73.2)		27 (67.5)		0.31	.57
Socioeconomic status						
Low	2 (4.9)					
Average	21 (51.2)		20 (50.0)		2.11	.34
High	18 (43.9)		20 (50.0)			

Note: EFD: group with executive function deficits; TD: group with typical development; academic: higher education more than 12 years of schooling including studies toward a degree or profession; socioeconomic status: low: under 7,000 ILS, average: 7,000–14,000 ILS, and high: more than 14,000 ILS for one month.

**Table 2:** Participants' inclusion criteria and additional measure for characterizing EFD profile.

Inclusion criteria	EFD group (*n* = 41)		TD group (*n* = 40)				
WISC-R95	*M* (SD)		*M* (SD)		*F*	*p*	*η*_*p*_^2^
Vocabulary	9.63 (2.43)		11.80 (2.69)		14.43	<.001	.15
Block design	11.20 (2.96)		13.38 (2.09)		14.56	<.001	.15

	*M* (SD)	Range	*M* (SD)	Range	*t*	*p*	
BRIEF-BRI	67.70 (9.72)	52-94	46.32 (8.15)	36-66	11.73	<.001	
BRIEF-MI	66.65 (6.34)	50-81	42.95 (6.13)	33-59	17.10	<.001	
BRIEF-GEC	69.09 (5.26)	58-80	43.82 (6.36)	33-55	19.44	<.001	

BRIEF subscales	*M* (SD)	Range	*M* (SD)	Range	*F*	*p*	*η*_*p*_^2^
Inhibition	63.00 (12.13)	40–100	45.92 (6.70)	40–63	61.01	<.001	.43
Shift	70.29 (11.04)	48–95	46.47 (6.86)	38–59	135.17	<.001	.63
Emotional control	68.68 (12.15)	45–91	47.05 (9.05)	36–64	82.17	<.001	.51
Initiation	62.21 (9.43)	44–79	42.47 (5.93)	35–56	126.35	<.001	.61
Working memory	67.44 (8.51)	49–82	43.65 (5.43)	36–57	223.32	<.001	.73
Plan/Org	67.41 (7.46)	47–82	43.77 (6.45)	37–63	231.87	<.001	.74
Organization of materials	59.46 (7.83)	40–70	46.60 (9.16)	34–66	46.17	<.001	.36
Monitor	63.65 (6.95)	48–82	42.87 (7.62)	33–58	164.36	<.001	.67

EFD profile	*M* (SD)	Range	*M* (SD)	Range	*t*	*p*	
BRIEF-SR-BRI	59.07 (12.18)	37-84	45.85 (10.36)	18-68	5.26	<.001	
BRIEF-SR-MI	58.71 (10.83)	31-81	43.35 (8.59)	22-63	7.07	<.001	
BRIEF-SR-GEC	59.39 (11.24)	33-84	45.10 (9.64)	19-67	6.14	<.001	

BRIEF-SR subscales	*M* (SD)	Range	*M* (SD)	Range	*F*	*p*	*η*_*p*_^2^
Inhibition	55.70 (11.07)	34–86	46.52 (8.29)	34–66	17.77	<.001	.18
Shift	58.60 (14.35)	32–91	45.85 (9.41)	32–78	22.25	<.001	.22
Emotional control	61.17 (10.96)	38–83	49.35 (9.98)	28–71	25.71	<.001	.25
Monitor	54.09 (10.10)	36–76	45.77 (8.51)	31–63	16.05	<.001	.17
Working memory	56.04 (11.39)	34–86	43.50 (7.57)	32–61	33.92	<.001	.30
Plan/Org	58.32 (10.89)	31–79	45.15 (10.49)	18–75	30.69	<.001	.28
Organization of materials	55.22 (11.54)	33–76	45.07 (8.00)	33–64	21.01	<.001	.21
Task completion	61.12 (11.00)	35–84	43.55 (8.08)	26–61	66.81	<.001	.46

WebNeuro domain	*M* (SD)		*M* (SD)		*F*	*p*	*η*_*p*_^2^
Memory	-0.59 (1.40)		.550 (.58)		23.15	<.001	.22
Attention	-1.09 (1.04)		.160 (.87)		34.93	<.001	.30
Executive	-0.57 (1.10)		.350 (.71)		20.16	<.001	.20

Note: EFD: group with executive function deficits; TD: group with typical development; *M*: mean; SD: standard deviation; WISC-95: Wechsler Intelligence Scale for Children; BRIEF: Behavior Rating Inventory of Executive Function.

**Table 3:** Differences between groups in the Children's Cooking Task assessment.

	EFD group (*N* = 41) *n* (%)	TD group (*N* = 40) *n* (%)	*χ* ^2^	*p*	
Have you performed cooking tasks in the past?	Yes: 36 (87.7)	Yes: 38 (95.7)	1.32	.25	
Are you allowed to experiment with home cooking activities?	Yes: 28 (68.3)	Yes: 32 (80.0)	1.47	.47	
Qualitative **rating**					
Goal achievement	No: 21 (51.2)	No: 6 (15.0)	11.90	<.010	
Dangerous behavior	Yes: 18 (43.9)	Yes: 2 (5.0)	16.47	<.001	
Intervention of adult	Yes: 19 (46.3)	Yes: 3 (7.5)	15.44	<.001	

	EFD group (*N* = 41)	TD group (*N* = 40)	Mann-Whitney test *Z*	*p*	*η*_*p*_^2^
	*M* [SD (min–max)]				
Task duration (min)	27.87 [12.22 (11–75)]	21.52[5.32 (14–47)]	2.91	<.001	
Total number of errors	44.41 [25.43 (12–135)]	9.98 [5.90 (2–28)]	7.33	<.001	.72
Descriptive analysis					
(1) omissions	2.54 [1.88 (0–8)]	0.73 [1.01 (0–4)]	5.10	<.001	.73
(2) additions	13.22 [9.15 (1–51)]	1.63 [1.39 (0–6)]	7.27	<.001	.72
(3) substitutions-sequences	5.32 [3.08 (0–11)]	1.75 [1.42 (0–4)]	5.37	<.001	.73
(4) estimation	5.63 [2.40 (0–10)]	1.00 [1.51 (0–6)]	6.84	<.001	.72
(5) commentary-question	18.88 [20.54 (0–113)]	4.90 [4.57 (0–21)]	4.03	<.001	.73
Neuropsychological analysis					
(N1) control errors	7.80 [4.56 (1–21)]	1.60 [1.80 (0–8)]	6.55	<.001	.72
(N2) context neglect	31.27 [21.24 (8–120)]	7.73 [5.12 (1-24)]	6.96	<.001	.72
(N3) environmental adherence	5.20 [3.87 (0–23)]	1.53 [1.46 (0–7)]	5.72	<.001	.73
(N4) purposeless action	6.22 [5.65 (0–20)]	0.58 [0.87 (0–4)]	6.37	<.001	.72
(N5) dependency	5.58 [4.18 (0–17)]	1.75 [1.41 (0–5)]	5.29	<.001	.73
(N6) inappropriate behavior	0.63 [1.09 (0–4)]	0.03 [0.15 (0–1)]	3.67	<.001	.73

Note: EFD: group with executive function deficits; TD: group with typical development; *M*: mean; SD: standard deviation.

**Table 4:** Discriminant function analysis structure matrix: predictors' loading value (descriptive and neuropsychological analyses).

Descriptive analysis	Function
(4) estimation errors	.81
(2) additions	.62
(3) substitutions-sequences	.52
(1) omissions	.42
(5) commentary-question	.28
Neuropsychological analysis	Function
(N1) control errors	.77
(N2) context neglect	.65
(N4) purposeless action	.59
(N5) dependency	.56
(N3) environmental adherence	.53
(N6) inappropriate behavior	.33

## Data Availability

The data used to support the findings of this study are restricted by the University of Haifa ethics committee (approval number 253/13) in order to protect patient privacy. Data are available from Yael Fogel (yfogel@gmail.com) for researchers who meet the criteria for access to confidential data.

## Appendix

### A. Types of Errors in the Cooking Task Analysis

As a first step, we classified errors participants performed during the CCT at a descriptive level:

*(1) Omission*. Any action or sequence of actions normally required to reach the goal that is either omitted or incompletely performed.

*Examples: forgetting a step or part of a step, forgetting to use soap when washing hands, forgetting to add an ingredient, and not asking the examiner to turn on the oven.*

*(2) Addition*. Any action or sequence of actions unnecessary for the completion of the task. Additions may be actions that could be part of the task that are then achieved with “detours” or actions that are irrelevant to the task.

*Examples: using distracter ingredients and adding extra ingredients to the recipe.*

*(3) Inversion-Substitution*. Any object mistake (misuse of one object for another) or use of an object *a priori* belonging to the correct category but inappropriate to the goal initially pursued (e.g., a dirty or dangerous object) or any sequence error.

*Examples: requesting to turn on the oven when the dough is finished and not at Step 1 as indicated in the recipe and preparing the dough in a small bowl.*

*(4) Estimation*. Poor estimation of the quantity of ingredients, the number or size of utensils, the place, or the time. Estimation errors are, so to speak, errors that have to do with amount, size, time, power, or place.

*Examples: pouring half a glass of oil instead of a full glass and pouring the flour directly into the glass and spilling part of the flour outside the glass.*

*(5) Commentary-Question*. Any question, remark, or joke to the examiners, such as asking how to perform an action or where to find an ingredient or a utensil, although the patient had been clearly instructed to act as if he or she were alone.

Next, we classified the errors in a second-step analysis, taking into account the neuropsychological mechanisms underlying the occurrence of errors.

*(1) Control Errors*. Inefficient monitoring of action or not respecting the instructions or the recipe. At the utmost, this lack of monitoring or insufficient control may cause failure to achieve the goal. This type of error highlights the role of EF in controlling the efficiency of the actions undertaken in relation to the goal.

*Examples: not respecting the quantity of an ingredient and adding the sparkling water to the cocktail glass until it overflows.*

*(2) Context Neglect*. Poor assessment of the environment or failure to respect the instructions or the frame defined for the task. This definition highlights the role of ER in the analysis and monitoring of environmental data in order to adapt one's behavior to the environment and better reach the goal.

*Examples: not washing hands, although they are covered in butter; playing football in the kitchen; licking fingers; and putting a spoon coated with dough into the flour bag.*

*(3) Environmental Adherence*. Action carried out because of the patient's adherence to an object or place. This type of error belongs to the symptoms typically described in a dysexecutive syndrome.

*Examples: breaking all the eggs of the box without stopping when the requested quantity is achieved and working on a crowded countertop or even on the recipe binder.*

*(4) Purposeless Actions and Displacements*. Action performed without any apparent goal, which does not contribute to pursuing the task; apathetic behavior. The EF intervene in the support of an activity to achieve a goal.

*Examples: walking around the table without performing any effective action and picking up an object and putting it down without using it.*

*(5) Dependency*. Any question or request for help by the participant, such as questioning the way to perform an action or to find an object. This type of error highlights the role of EF in the search and implementation of backup strategies when the solution to a problem is not directly reachable, as well as the structuring role of the examiner during neuropsychological testing.

*Examples: “how many eggs in the cake?” or “what is a stick of butter?” or, pointing to the spoon, “do I use this to mix the dough?”*

*(6) Inappropriate Behavior*. Any socially inappropriate or dangerous behavior. These errors highlight the role of EF in the behavioral regulation and adaptation in relation to the environment.

*Examples: asking the examiner to taste the dough, making or answering a phone call while performing the task, and attempting to remove the hot mold from the oven alone.*

## References

[1] Otero T. M., Barker L. A., Naglieri J. A. (2014). Executive function treatment and intervention in schools. *Applied Neuropsychology: Child*.

[2] Anderson P. (2002). Assessment and development of executive function (EF) during childhood. *Child Neuropsychology*.

[3] Barkley R. A. (1997). Behavioral inhibition, sustained attention, and executive functions: constructing a unifying theory of ADHD. *Psychological Bulletin*.

[4] Wallisch A., Little L. M., Dean E., Dunn W. (2017). Executive function measures for children: a scoping review of ecological validity. *OTJR: Occupation, Participation and Health*.

[5] Best J. R., Miller P. H., Jones L. L. (2009). Executive functions after age 5: changes and correlates. *Developmental Review*.

[6] Diamond A. (2013). Executive functions. *Annual Review of Psychology*.

[7] Cramm H., Krupa T., Missiuna C., Lysaght R. M., Parker K. C. (2013). Broadening the occupational therapy toolkit: an executive functioning lens for occupational therapy with children and youth. *The American Journal of Occupational Therapy*.

[8] De Luca C. R., Leventer R. J., Anderson V., Jacobs R., Anderson P. J. (2010). Developmental trajectories of executive functions across the lifespan. *Executive Functions and the Frontal Lobes: A Lifespan Perspective*.

[9] Crone E. A., Dahl R. E. (2012). Understanding adolescence as a period of social-affective engagement and goal flexibility. *Nature Reviews Neuroscience*.

[10] Josman N., Rosenblum S., Katz N., Toglia J. (2018). A metacognitive model for children with neurodevelopmental disabilities. *Cognition, Occupation, and Participation across the Life Span: Neuroscience, Neurorehabilitation, and Models for Intervention in Occupational Therapy*.

[11] Silverstein S. M., Berten S., Olson P. (2007). Development and validation of a world-wide-web-based neurocognitive assessment battery: WebNeuro. *Behavior Research Methods*.

[12] Bernardi M., Leonard H. C., Hill E. L., Botting N., Henry L. A. (2018). Executive functions in children with developmental coordination disorder: a 2-year follow-up study. *Developmental Medicine and Child Neurology*.

[13] Meltzer L. (2018). *Executive Function in Education: From Theory to Practice*.

[14] Weiner N. W., Toglia J., Berg C. (2012). Weekly calendar planning activity (WCPA): a performance-based assessment of executive function piloted with at-risk adolescents. *The American Journal of Occupational Therapy*.

[15] Barkley R. A. (1991). The ecological validity of laboratory and analogue assessment methods of ADHD symptoms. *Journal of Abnormal Child Psychology*.

[16] Chaytor N., Schmitter-Edgecombe M. (2003). The ecological validity of neuropsychological tests: a review of the literature on everyday cognitive skills. *Neuropsychology Review*.

[17] Chevignard M. P., Soo C., Galvin J., Catroppa C., Eren S. (2012). Ecological assessment of cognitive functions in children with acquired brain injury: a systematic review. *Brain Injury*.

[18] Gioia G. A., Isquith P. K., Guy S. C., Kenworthy L. (2000). *Behavior rating inventory of executive function: BRIEF*.

[19] Gioia G. A., Isquith P. K., Guy S. C., Kenworthy L. (2016). *Behavior Rating Inventory of Executive Function (BRIEF-2)*.

[20] Guy S. C., Isquith P. K., Gioia G. A. (2004). *Behavior Rating Inventory of Executive Function: Self-Report Version Professional Manual*.

[21] Emslie H., Wilson F. C., Burden V., Nimmo-Smith I., Wilson B. A. (2003). *Behavioural Assessment of the Dysexecutive Syndrome in Children (BADS-C)*.

[22] Josman N., Goffer A., Rosenblum S. (2010). Development and standardization of a "Do-Eat" activity of daily living performance test for children. *The American Journal of Occupational Therapy*.

[23] Rosenblum S., Frisch C., Deutsh-Castel T., Josman N. (2015). Daily functioning profile of children with attention deficit hyperactive disorder: a pilot study using an ecological assessment. *Neuropsychological Rehabilitation*.

[24] Toglia J. (2010). *Weekly Calendar Planning Activity*.

[25] Chevignard M. P., Servant V., Mariller A., Abada G., Pradat-Diehl P., Laurent-Vannier A. (2009). Assessment of executive functioning in children after TBI with a naturalistic open-ended task: a pilot study. *Developmental Neurorehabilitation*.

[26] Chevignard M. P., Catroppa C., Galvin J., Anderson V. (2010). Development and evaluation of an ecological task to assess executive functioning post childhood TBI: the Children's Cooking Task. *Brain Impairment*.

[27] Toussaint-Thorin M., Marchal F., Benkhaled O., Pradat-Diehl P., Boyer F. C., Chevignard M. (2013). Executive functions of children with developmental dyspraxia: assessment combining neuropsychological and ecological tests. *Annals of Physical and Rehabilitation Medicine*.

[28] Lezak M. D., Le Gall D., Aubin G. (1994). Evaluation des fonctions exécutives lors des atteintes des lobes frontaux. *Revue de Neuropsychologie*.

[29] Chevignard M., Pillon B., Pradat-Diehl P. (2000). An ecological approach to planning dysfunction: script execution. *Cortex*.

[30] Chevignard M. P., Taillefer C., Picq C., Poncet F., Noulhiane M., Pradat-Diehl P. (2008). Ecological assessment of the dysexecutive syndrome using execution of a cooking task. *Neuropsychological Rehabilitation*.

[31] Baum C., Christiansen C., Bass J., Christiansen C., Baum C., Bass J. (2015). Person-Environment-Occupational Performance (PEOP) model. *Occupational Therapy: Performance, Participation, Well-Being*.

[32] Lamberts K. F., Evans J. J., Spikman J. M. (2010). A real-life, ecologically valid test of executive functioning: the executive secretarial task. *Journal of Clinical and Experimental Neuropsychology*.

[33] Toglia J., Johnston M. V., Goverover Y., Dain B. (2010). A multicontext approach to promoting transfer of strategy use and self regulation after brain injury: an exploratory study. *Brain Injury*.

[34] Downes M., Berg C., Kirkham F. J., Kischkel L., McMurray I., de Haan M. (2018). Task utility and norms for the Preschool Executive Task Assessment (PETA). *Child Neuropsychology*.

[35] Wechsler D. (1998). *Wechsler Scale for Intelligence: Hebrew Version*.

[36] Sattler J. M. (2008). *Assessment of Children: Cognitive Applications*.

[37] Bental B., Tirosh E. (2007). The relationship between attention, executive functions and reading domain abilities in attention deficit hyperactivity disorder and reading disorder: a comparative study. *Journal of Child Psychology and Psychiatry*.

[38] Loo S. K., Humphrey L. A., Tapio T. (2007). Executive functioning among Finnish adolescents with attention-deficit/hyperactivity disorder. *Journal of the American Academy of Child and Adolescent Psychiatry*.

[39] Riggs N. R., Shin H. S., Unger J. B., Spruijt-Metz D., Pentz M. A. (2014). Prospective associations between bilingualism and executive function in Latino children: sustained effects while controlling for biculturalism. *Journal of Immigrant and Minority Health*.

[40] Riggs N. R., Spruijt-Metz D., Sakuma K. L., Chou C. P., Pentz M. A. (2010). Executive cognitive function and food intake in children. *Journal of Nutrition Education and Behavior*.

[41] Poncet F., Krasny-Pacini A., Alzieu C., Servant V., Taillefer C., Chevignard M. (2015). *Children’s Cooking Task Manual (English version1)*.

[42] Hirsh R. (2016). *Executive functions among adolescents with neurodevelopmental disabilities: establish discriminate validity and concurrent validity for the children cooking task (Children’s Cooking Task) tool [master's thesis]*.

[43] Faul F., Erdfelder E., Buchner A., Lang A. G. (2009). Statistical power analyses using G∗Power 3.1: tests for correlation and regression analyses. *Behavior Research Methods*.

[44] Faul F., Erdfelder E., Lang A. G., Buchner A. (2007). G∗Power 3: a flexible statistical power analysis program for the social, behavioral, and biomedical sciences. *Behavior Research Methods*.

[45] Toplak M. E., West R. F., Stanovich K. E. (2013). Practitioner review: do performance-based measures and ratings of executive function assess the same construct?. *Journal of Child Psychology and Psychiatry*.

[46] Becker L. A. (2000). *Effect size (ES)*.

[47] McAuley T., Chen S., Goos L., Schachar R., Crosbie J. (2010). Is the behavior rating inventory of executive function more strongly associated with measures of impairment or executive function?. *Journal of the International Neuropsychological Society*.

[48] Rosenblum S. (2012). Validity and reliability of the time organisation and participation scale (TOPS). *Neuropsychological Rehabilitation*.

[49] Zentall S. S., Harper G. W., Stormont-Spurgin M. (1993). Children with hyperactivity and their organizational abilities. *The Journal of Educational Research*.

[50] Ma Y., Wang E., yuan T., Zhao G. (2016). Location negative priming effects in children with developmental dyslexia: an event-related potential study. *Research in Developmental Disabilities*.

[51] Krieger V., Amador-Campos J. A. (2018). Assessment of executive function in ADHD adolescents: contribution of performance tests and rating scales. *Child Neuropsychology*.

[52] Isquith P. K., Crawford J. S., Espy K. A., Gioia G. A. (2005). Assessment of executive function in preschool-aged children. *Mental Retardation and Developmental Disabilities Research Reviews*.

[53] Shimoni M., Engel-Yeger B., Tirosh E. (2012). Executive dysfunctions among boys with attention deficit hyperactivity disorder (ADHD): performance-based test and parents report. *Research in Developmental Disabilities*.

[54] Josman N., Meyer S. (2019). Conceptualisation and use of executive functions in paediatrics: a scoping review of occupational therapy literature. *Australian Occupational Therapy Journal*.

[55] Toglia J., Katz N., Toglia J. (2018). The dynamic interactional model and the multicontext approach. *Cognition, Occupation, and Participation across the Life Span: Neuroscience, Neurorehabilitation, and Models for Intervention in Occupational Therapy*.

[56] Ownsworth T., Fleming J., Tate R. (2017). Do people with severe traumatic brain injury benefit from making errors? A randomized controlled trial of error-based and errorless learning. *Neurorehabilitation and Neural Repair*.

